# Supplementary material for: Protocol for a systematic review assessing ambulatory vital sign monitoring impact on deterioration detection and related clinical outcomes in hospitalised patients
Source: BMJ Open. 2021 May 18;11(5):e047715. doi: 10.1136/bmjopen-2020-047715 (PMC8130745; doi:10.1136/bmjopen-2020-047715)
Supplement: Supplementary data [file bmjopen-2020-047715supp001.pdf]

Appendix 1 – Example Medline search terms

Database: Medline (Ovid MEDLINE® Epub Ahead of Print, In-Process & Other Non-Indexed Citations, Ovid MEDLINE® Daily and Ovid MEDLINE®) 1946 to present

Search Strategy: (27/08/20)

----

1 exp Monitoring, Ambulatory/ (28543)

2 ambulatory.tw. (78329)

3 exp Wearable Electronic Devices/ (11822)

4 wearable.tw. (12014)

5 (body adj3 borne).tw. (72)

6 (head mount* adj3 (display* or device* or system*)).tw. (1101)

7 (head-up adj3 (display* or device* or system*)).tw. (153)

8 (head-worn adj3 (display* or device* or system*)).tw. (31)

9 (patch or patches).tw. (106471)

10 Adhesives/ (6682)

11 adhesive*.tw. (57587)

12 (sticker* or stick or sticks).tw. (12250)

13 (watch or watches).tw. (9028)

14 smartwatch*.tw. (309)

15 telemonitor*.tw. (1516)

16 tele monitor*.tw. (146)

17 exp Telemetry/ (13327)

18 telemet*.tw. (9378)

19 clothing/ or shoes/ (15121)

20 cloth*.tw. (16130)

Areia C, *et al*. *BMJ Open* 2021; 11:e047715. doi: 10.1136/bmjopen-2020-047715

21 exp Textiles/ (6392)

22 textile*.tw. (10792)

23 exp Telemedicine/ (29383)

24 telemedicine*.tw. (10894)

25 exp Biosensing Techniques/ (54829)

26 biosens*.tw. (37047)

27 glove*.tw. (10632)

28 shoe*.tw. (9295)

29 accessor*.tw. (44268)

30 Wrist/ (9011)

31 wrist*.tw. (38559)

32 necklace*.tw. (904)

33 (belt or belts).tw. (12916)

34 armband*.tw. (590)

35 (shirt or shirts).tw. (1067)

36 contactless.tw. (1731)

37 cableless.tw. (8)

38 contact sensor*.tw. (184)

39 unobtrusive.tw. (1596)

40 remote.tw. (69973)

41 Wireless Technology/ (3587)

42 wireless*.tw. (14654)

43 non contact.tw. (5281)

44 noncontact.tw. (4723)

45 without wires.tw. (21)

46 non-restrict*.tw. (555)

47 unrestrict*.tw. (8566)

48 Automation/ (18179)

49 automat*.tw. (229850)

50 webcam*.tw. (397)

51 web-cam*.tw. (175)

52 video*.tw. (126640)

53 2 or 3 or 4 or 5 or 6 or 7 or 8 or 9 or 10 or 11 or 12 or 13 or 14 or 15 or 16 or 17 or 18 or 19 or 20 or 21 or 22 or 23 or 24 or 25 or 26 or 27 or 28 or 29 or 30 or 31 or 32 or 33 or 34 or 35 or 36 or 37 or 38 or 39 or 40 or 41 or 42 or 43 or 44 or 45 or 46 or 47 or 48 or 49 or 50 or 51 or 52 (979503)

54 Monitoring, Physiologic/mt [Methods] (18136)

55 monitor*.tw. (804541)

56 Oximetry/ (13024)

57 oximet*.tw. (13397)

58 tracker*.tw. (4568)

59 Radar/ (1203)

60 radar*.tw. (5333)

61 Ballistocardiography/ (2049)

62 ballisto*.tw. (1831)

63 exp Accelerometry/ (8929)

64 accelerom*.tw. (16743)

65 gyro*.tw. (4773)

66 Photoplethysmography/ (1987)

67 ppg.tw. (3458)

68 photople*.tw. (3299)

69 videopleth*.tw. (4)

70 video pleth*.tw. (6)

71 (photo* adj3 pleth*).tw. (339)

72 Thermography/ (7571)

73 thermograph*.tw. (6133)

74 Infrared Rays/ (13281)

75 infrared*.tw. (137632)

76 thermal.tw. (200146)

77 thermistor*.tw. (1748)

78 thermoresister*.tw. (0)

79 camera*.tw. (45491)

80 wavelet analysis/ (2189)

81 wavelet*.tw. (11286)

82 waveform*.tw. (28510)

83 ambient light*.tw. (1886)

84 piezoelectric*.tw. (10492)

85 piezo electric*.tw. (342)

86 Electric Impedance/ (17145)

87 impedan*.tw. (45070)

88 exp Electrocardiography/ (204339)

89 electrocar*.tw. (88334)

90 (ecg or ecgs).tw. (65572)

91 (ekg or ekgs).tw. (3081)

92 electrom*.tw. (97510)

93 electrog*.tw. (20290)

94 exp Electrodes/ (130099)

95 electrode*.tw. (161247)

96 SensiumVitals.tw. (8)

97 IntelliVue Guardian.tw. (0)

98 VitalPatch.tw. (2)

99 Vitalsolutions.tw. (0)

100 Guardian Angel.tw. (74)

101 Visi Mobile.tw. (6)

102 Current health.tw. (4290)

103 Snap40.tw. (1)

104 Multi-vital ECG patch.tw. (0)

105 Caretaker.tw. (1456)

106 Life scope G3.tw. (0)

107 Biostamp.tw. (4)

108 TAGECG.tw. (0)

109 ZioXT patch.tw. (0)

110 ZioAT patch.tw. (0)

111 Cardea solo.tw. (0)

112 Peerbridge Cor.tw. (0)

113 Radius-7.tw. (24)

114 Radius-PPG.tw. (0)

115 WristOx2.tw. (4)

116 Lifesync.tw. (1)

117 Ariatele.tw. (0)

118 54 or 55 or 56 or 57 or 58 or 59 or 60 or 61 or 62 or 63 or 64 or 65 or 66 or 67 or 68 or 69 or 70 or 71 or 72 or 73 or 74 or 75 or 76 or 77 or 78 or 79 or 80 or 81 or 82 or 83 or 84 or 85 or 86 or 87 or 88 or 89 or 90 or 91 or 92 or 93 or 94 or 95 or 96 or 97 or 98 or 99 or 100 or 101 or 102 or 103 or 104 or 105 or 106 or 107 or 108 or 109 or 110 or 111 or 112 or 113 or 114 or 115 or 116 or 117 (1802638)

119 53 and 118 (177463)

120 1 or 119 (194037)

Areia C, *et al. BMJ Open* 2021; 11:e047715. doi: 10.1136/bmjopen-2020-047715

121 vital signs/ or blood pressure/ or body temperature/ or heart rate/ or respiratory rate/ (410477)

122 heart rate*.tw. (159199)

123 pulse rate*.tw. (7614)

124 pulse*.tw. (256042)

125 respiratory rate*.tw. (14992)

126 breathing rate*.tw. (1234)

127 respiration.tw. (67094)

128 breathing.tw. (71424)

129 blood pressure.tw. (294342)

130 systolic.tw. (168584)

131 diastolic.tw. (124777)

132 perfusion.tw. (159943)

133 spo2.tw. (5098)

134 oxygenation.tw. (52011)

135 oxygen saturation.tw. (26170)

136 temperature*.tw. (651316)

137 exp Fever/ (43448)

138 fever*.tw. (171789)

139 hypertherm*.tw. (35327)

140 pyrex*.tw. (5346)

141 Hypothermia/ (13917)

142 hypotherm*.tw. (41950)

143 apyrex*.tw. (210)

144 a-pyrex*.tw. (173)

145 vital sign*.tw. (14891)

146 vitals.tw. (500)

147 vital param*.tw. (1004)

148 vital function*.tw. (2790)

149 early warning score/ (91)

150 (early warning adj3 scor*).tw. (1049)

151 (early warning adj3 system*).tw. (2134)

152 mews.tw. (261)

153 ews.tw. (1986)

154 ewss.tw. (73)

155 (track and trigger).tw. (331)

156 tts.tw. (2485)

157 risk assessment tool*.tw. (3049)

158 febrile.tw. (35432)

159 afebrile.tw. (2897)

160 a-febrile.tw. (2540)

161 121 or 122 or 123 or 124 or 125 or 126 or 127 or 128 or 129 or 130 or 131 or 132 or 133 or 134 or 135 or 136 or 137 or 138 or 139 or 140 or 141 or 142 or 143 or 144 or 145 or 146 or 147 or 148 or 149 or 150 or 151 or 152 or 153 or 154 or 155 or 156 or 157 or 158 or 159 or 160 (2068958)

162 Hospitalization/ (108262)

163 hospital*.tw. (1288499)

164 inhospital.tw. (1750)

165 in-hospital.tw. (89036)

166 admitted.tw. (206110)

167 inpatient*.tw. (108524)

168 surgical.tw. (968814)

169 pacu.tw. (2502)

170 post anaesthesia care unit*.tw. (328)

171 post anesthesia care unit*.tw. (740)

172 postanaesthesia care unit*.tw. (192)

173 postanesthesia care unit*.tw. (1900)

174 icu.tw. (56820)

175 exp Critical Care/ (58129)

176 exp Intensive Care Units/ (85074)

177 intensive care.tw. (144066)

178 itu.tw. (869)

179 aicu.tw. (45)

180 icus.tw. (10393)

181 itus.tw. (43)

182 aicus.tw. (7)

183 pacus.tw. (82)

184 critical care.tw. (27729)

185 (ward or wards).tw. (59542)

186 Triage/ (11887)

187 triage*.tw. (18767)

188 Emergencies/ (40466)

189 (emergency or emergencies).tw. (270872)

190 Recovery Room/ (1297)

191 recovery room*.tw. (3323)

192 adolescent, hospitalized/ or child, hospitalized/ or inpatients/ (29074)

193 162 or 163 or 164 or 165 or 166 or 167 or 168 or 169 or 170 or 171 or 172 or 173 or 174 or 175 or 176 or 177 or 178 or 179 or 180 or 181 or 182 or 183 or 184 or 185 or 186 or 187 or 188 or 189 or 190 or 191 or 192 (2563179)

194 120 and 161 and 193 (5355)
