# Supplementary material for: Protocol for a systematic review assessing ambulatory vital sign monitoring impact on deterioration detection and related clinical outcomes in hospitalised patients
Source: BMJ Open. 2021 May 18;11(5):e047715. doi: 10.1136/bmjopen-2020-047715 (PMC8130745; doi:10.1136/bmjopen-2020-047715)
Supplement: Author's manuscript [file bmjopen-2020-047715.draft_revisions.pdf]

BMJ Open is committed to open peer review. As part of this commitment we make the peer review history of every article we publish publicly available.

When an article is published we post the peer reviewers' comments and the authors' responses online. We also post the versions of the paper that were used during peer review. These are the versions that the peer review comments apply to.

The versions of the paper that follow are the versions that were submitted during the peer review process. They are not the versions of record or the final published versions. They should not be cited or distributed as the published version of this manuscript.

BMJ Open is an open access journal and the full, final, typeset and author-corrected version of record of the manuscript is available on our site with no access controls, subscription charges or pay-per-view fees (http://bmjopen.bmj.com).

If you have any questions on BMJ Open's open peer review process please email info.bmjopen@bmj.com

BMJ Open

Protocol for a systematic review assessing ambulatory vital sign monitoring impact on deterioration detection and related clinical outcomes in hospitalised patients.

Journal:	*BMJ Open*
Manuscript ID	bmjopen-2020-047715
Article Type:	Protocol
Date Submitted by the Author:	07-Dec-2020
Complete List of Authors:	Areia, Carlos; University of Oxford, Nuffield Department of Clinical Neurosciences<br>Vollam, Sarah; University of Oxford, Nuffield Department of Clinical Neurosciences<br>Young, Louise; University of Oxford, Nuffield Department of Clinical Neurosciences<br>Biggs, Christopher; University of Oxford, Nuffield Department of Clinical Neurosciences<br>Pimentel, Marco; University of Oxford, Institute of Biomedical Engineering, Department of Engineering Science<br>Santos, Mauro; University of Oxford, Institute of Biomedical Engineering, Department of Engineering Science<br>Thurley, Neal; University of Oxford, Bodleian Health Care Libraries<br>Gerry, Stephen; University of Oxford, Centre for Statistics in Medicine<br>Tarassenko, Lionel; University of Oxford, Institute of Biomedical Engineering, Department of Engineering Science<br>Watkinson, Peter; University of Oxford, Nuffield Department of Clinical Neurosciences
Keywords:	Health informatics < BIOTECHNOLOGY & BIOINFORMATICS, Telemedicine < BIOTECHNOLOGY & BIOINFORMATICS, ANAESTHETICS, BIOTECHNOLOGY & BIOINFORMATICS

SCHOLARONE™
Manuscripts

I, the Submitting Author has the right to grant and does grant on behalf of all authors of the Work (as defined in the below author licence), an exclusive licence and/or a non-exclusive licence for contributions from authors who are: i) UK Crown employees; ii) where BMJ has agreed a CC-BY licence shall apply, and/or iii) in accordance with the terms applicable for US Federal Government officers or employees acting as part of their official duties; on a worldwide, perpetual, irrevocable, royalty-free basis to BMJ Publishing Group Ltd ("BMJ") its licensees and where the relevant Journal is co-owned by BMJ to the co-owners of the Journal, to publish the Work in this journal and any other BMJ products and to exploit all rights, as set out in our licence.

The Submitting Author accepts and understands that any supply made under these terms is made by BMJ to the Submitting Author unless you are acting as an employee on behalf of your employer or a postgraduate student of an affiliated institution which is paying any applicable article publishing charge ("APC") for Open Access articles. Where the Submitting Author wishes to make the Work available on an Open Access basis (and intends to pay the relevant APC), the terms of reuse of such Open Access shall be governed by a Creative Commons licence – details of these licences and which Creative Commons licence will apply to this Work are set out in our licence referred to above.

Other than as permitted in any relevant BMJ Author's Self Archiving Policies, I confirm this Work has not been accepted for publication elsewhere, is not being considered for publication elsewhere and does not duplicate material already published. I confirm all authors consent to publication of this Work and authorise the granting of this licence.

For peer review only

Title: Protocol for a systematic review assessing ambulatory vital sign monitoring impact on deterioration detection and related clinical outcomes in hospitalised patients.

Authors:

Carlos Areia[1]

Sarah Vollam[1]

Louise Young[1]

Christopher Biggs[1]

Marco Pimentel[2]

Mauro Santos[2]

Neal Thurley[3]

Stephen Gerry [4]

Lionel Tarassenko[2]

Peter Watkinson[1]

[1] Critical Care Research Group, Nuffield Department of Clinical Neurosciences, University of Oxford, Oxford, Oxfordshire, UK

[2] Institute of Biomedical Engineering, Department of Engineering Science, University of Oxford, Oxford, Oxfordshire, UK

[3] Bodleian Health Care Libraries, University of Oxford, Oxford, Oxfordshire, UK

[4] Centre for Statistics in Medicine, University of Oxford, Oxford, Oxfordshire, UK

Corresponding author:

Carlos Areia

Critical Care Research Group, Nuffield Department of Clinical Neurosciences, University of Oxford

Kadoorie Research Centre, John Radcliffe Hospital, Oxford, Oxfordshire, UK

OX3 9DU

carlos.morgadoareia@ndcn.ox.ac.uk

For peer review only - http://bmjopen.bmj.com/site/about/guidelines.xhtml

Word count - 3300

For peer review only - http://bmjopen.bmj.com/site/about/guidelines.xhtml

Abstract

Introduction

Ambulatory monitoring systems (AMS) can facilitate early detection of clinical deterioration, and have the potential to improve hospitalised patient outcomes. The objective of this systematic review is to assess the impact of vital signs monitoring on detection of deterioration and related outcomes in hospitalised patients using ambulatory monitoring systems, in comparison with standard care.

Methods and analysis

A systematic search will be conducted in MEDLINE, Embase, CINAHL, Cochrane Database of Systematic Reviews, CENTRAL and Health Technology Assessment databases, as well as grey literature. Search results will be reviewed in accordance with the Preferred Reporting Items for Systematic Review and Meta-Analysis checklist for systematic reviews. Studies comparing the use of ambulatory monitoring devices against standard care for deterioration detection and related clinical outcomes in hospitalised patients will be included and further clinical and other outcomes will also be explored. Deterioration related outcomes may include (but not limited to) unplanned intensive care admissions, rapid response team activation and unscheduled emergency interventions, as defined by the included studies. Two reviewers will independently extract study data and assess the quality and risk of bias of included studies. Where possible, a meta-analysis will be conducted and quantitative results presented. Alternatively, a narrative synthesis will be reported.

Ethics and Dissemination

Ethical approval is not required for this study as no primary data will be collected. This study is part of our virtual High Dependency Unit (vHDU) project and will be disseminated through peer-reviewed publications, public and scientific conference presentations.

PROSPERO Registration number

CRD42020188633

Keywords

ambulatory monitoring; deterioration detection; wearables; vital signs;

Strength and limitations of the study

- This is the first systematic review assessing the effect of ambulatory monitoring devices on deterioration detection and related clinical outcomes in comparison with standard care.
- This review may provide an indication of the impact of ambulatory monitoring devices use in the hospital environment.
- We will review clinical trial registries and report the proportion of published studies against registered.

For peer review only - http://bmjopen.bmj.com/site/about/guidelines.xhtml

Background

The utilisation of physiological early warning scoring (EWS) systems which monitor "standard" vital signs, including pulse rate, respiratory rate, blood pressure, oxygen saturations and temperature) remains current practice, coupled with a graded response such as referral for a senior review or increasing monitoring frequency [1]. This frequency of observations is generally guided by the clinical condition of the patient. However, intermittent measurement of vital signs (including application of devices and documentation) can be time consuming for healthcare professionals [2] and therefore, the desired monitoring frequency of is often not achieved [3]. This is identified as a limitation of intermittent monitoring systems, as they are dependent on the frequency of physiological monitoring observations [4]; of more concern is that even when ideal frequency is achieved, patients might deteriorate between observation sets [5].

Higher risk patients are often continuously monitored, improving early detection of deterioration [2]. However, in the UK, continuous monitoring is not commonly used in the ward environment [6]. In a systematic review, Downey and colleagues (2018) suggested that continuous vital sign monitoring outside the critical care setting may be feasible and has the potential to improve patient outcomes when compared to intermittent monitoring [5]. Another study suggested that implementing continuous monitoring in surgical wards was also cost effective [8]. On the other hand, clinical staff interviews indicate that despite the benefits and potential to increase the timely detection of patient deterioration, limitations in continuous vital sign monitoring technology can pose a barrier to implementation [2]. One of the main suggested reasons identified was invasiveness and restricted mobility [6,7].

As a response to current limitations in healthcare monitoring, companies are extending the capabilities of commercially available wearable ambulatory vital sign monitoring systems [9]. These non-wired monitors may provide an alternative continuous monitoring system, affording patients more mobility, less discomfort, reduce nursing time and improve the early detection of abnormal physiological parameters [10]. Nevertheless, a major barrier to the clinical implementation of these systems is their unproven reliability, efficiency and data fidelity [9]. Another challenge is the potential detrimental effect of motion on the data derived from such monitoring systems, for example, motion is known to affect the accuracy of pulse oximetry readings [11]. This was seen throughout a clinically relevant range of measurements, with less accuracy at lower arterial oxygen saturations, which is clearly highly undesirable in clinical practice.

A recent systematic review and meta-analysis assessed the impact of multi-parameter continuous non-invasive monitoring in hospital wards, suggesting that patients monitored in this way had a 39% decreased mortality risk when compared to those receiving intermittent monitoring; as well as a trend of reduced intensive care unit (ICU) transfer, rapid response team activation and hospital length of stay [12]. However, this review focused on non-invasive continuous monitoring and excluded neonatal, paediatric, obstetric, ICU and high-risk acute patients. Another recent review analysed the validation, feasibility, clinical outcomes and costs of 13 different wearable devices and concluded that these were predominantly in the validation and feasibility testing phases [13], highlighting the lack of studies exploring clinical outcomes. However, this review focused on recent wearable devices, and only including studies between 2009-2019. Furthermore, while there are many devices claiming the ability to safely monitor patients at risk of deterioration [14], evidence assessing the impact of ambulatory monitoring systems in the ward environment remains inconclusive, limiting implementation and clinical use [14].

For peer review only - http://bmjopen.bmj.com/site/about/guidelines.xhtml

Aims and objectives

The objective of this systematic review is to assess the impact of vital sign monitoring in the deterioration detection and related clinical outcomes of hospitalised patients using ambulatory monitoring systems in comparison with standard care.

Methods and Analysis

This systematic review was registered with the International Prospective Register of Systematic Reviews (PROSPERO) on the 10th July 2020 (registration number CRD42020188633). This review will be conducted in accordance with the Preferred Reporting Items for Systematic Reviews and Meta-Analyses (PRISMA) checklist [15]. This protocol is in accordance with the PRISMA-Protocol (PRISMA-P) statement checklist [16].

Methodological considerations

Inclusion and exclusion criteria

Types of studies

Randomised controlled trials, interventional studies, observational studies (including case-control or cohort studies) and pilot studies will be included. Retrospective studies that comply with the proposed outcomes and eligibility criteria will be considered. To minimise publication bias, unpublished studies will also be included. There will be no date or language restrictions as every effort will be attempted to translate a non-English article.

Systematic, narrative and scoping reviews will be excluded, but we may use relevant reviews to facilitate identification of original data where appropriate. Diagnostic accuracy and validation studies purely assessing device accuracy or reliability will also be excluded. However, where possible for the included studies, we will extract appropriate diagnostic accuracy metrics as a secondary outcome.

Types of participants

We will include any studies conducted in the hospital environment. Although we are only including data from admitted patients, there are no restrictions in minimum length of stay or monitoring period. Studies conducted in healthy volunteers or non-hospitalised patients will be excluded.

Interventions

Studies that include non- or minimally invasive ambulatory systems not part of standard care will be included. As per it's definition, ambulatory monitoring systems (AMS) should not be restrictive or pose a barrier to patient mobility. To be included, AMS should monitor one or more vital sign (heart rate, respiratory rate, temperature, blood pressure or oxygen saturation) either continuously or intermittently (eg. sending streams of data every 5 minutes) and measurements should be automated, therefore requiring frequent manual input from the clinical staff. These systems can additionally:

- include one or more ambulatory monitoring devices;

For peer review only - http://bmjopen.bmj.com/site/about/guidelines.xhtml

- provide any other measurements that might be considered for analysis (such as ECG, PPG, accelerometry, etc…) as long as the device measures at least one vital sign.

Comparators

For our primary outcome we will include studies that compare the ambulatory monitoring systems to standard care. Standard ward care can include (but is not limited to) the following measurements:

- **Heart rate:** Manual pulse rate count, spot checks using blood pressure machine/pulse oximeter, derived from PPG, ECG or arterial pressure waveforms.
- **Respiratory rate:** Manual breathing rate count, derived from capnography or ECG waveform.
- **Blood pressure:** Blood pressure cuff reading, derived from arterial pressure waveform.
- **Oxygen saturation:** Pulse oximetry, arterial blood gas.
- **Temperature:** oropharyngeal, nasopharyngeal, rectal and skin thermometers.

If enough studies are available, we will do a sub-group analysis according to the type of standard monitoring (intermittent manual vital sign measurement or automated measurements) as these may vary between studies and wards (eg. intensive care units using standard continuous wired monitoring).

As a secondary outcome, we will also include studies comparing different devices, if they comply with the eligibility criteria.

Outcomes

We will include studies measuring deterioration detection and related clinical outcomes. If relevant, we will also include studies reporting other clinical outcomes in hospitalised patients similar to other reviews [12]. It is likely there may be a great variety of outcomes measurements not only on patient outcomes but also in devices reliability and efficacy. Depending on the quantity and quality of papers fulfilling eligibility criteria and being included in the review; we may report a qualitative synthesis instead of a quantitative analysis.

Primary Outcomes

We will aim to compare the ambulatory monitoring systems with standard care in regard to deterioration detection and related clinical outcomes metrics. We will be flexible, as we predict studies may use different outcomes and time frames. These can include (but are not limited to):

1- ICU admission
2- Rapid response team (RRT) activation
3- Complications and adverse events
4- Emergency/unscheduled interventions (eg. cardio pulmonary resuscitation, surgery, antibiotics, etc…)

For peer review only - http://bmjopen.bmj.com/site/about/guidelines.xhtml

Secondary Outcomes

As a secondary outcome, we will report any other relevant patient related outcomes (eg. mortality, length of stay and acute changes in therapy/medication). Similarly, these metrics are likely to vary between studies so there will be no pre-determined criteria for inclusion at this point.

If enough data are available, we will also report device accuracy and reliability measurements, as well as the level of agreement between ambulatory monitoring systems and the standard care comparator (where available).

Exploratory Outcomes

We will narratively report alerting systems used and explore their impact on clinical outcomes for the included studies, where reported. This may include type of early warning score, alert thresholds used for each vital sign and other relevant alert parameters or information.

We will also perform a clinical trial registry search and narratively report the proportion and characteristics of eligible registered studies against the number of results published (linking the respective registered studies end date and timelines with published reports).

Information sources

Electronic searches

Searches will be performed in multiple databases including MEDLINE Ovid (including Epub Ahead of Print and In-Process & Other Non-Indexed Citations), Embase (Ovid), CINAHL (EBSCO) and Cochrane Database of Systematic Reviews (Cochrane Library, Wiley), Cochrane Central Register of Controlled Trials (CENTRAL) (Cochrane Library, Wiley) and Health Technology Assessment (HTA) database via https://www.crd.york.ac.uk/CRDWeb/.

OpenGrey http://www.opengrey.eu/ will be searched for any unpublished grey literature. Given the popular culture/commercial applications of the technology, Google Scholar via https://scholar.google.com/ will also be searched. Additionally, we will also seek to identify eligible pre-prints using medRxiv via https://www.medrxiv.org/ . If relevant papers are identified through included studies references, these may also be included. Finally, we will also search trial registries such as ClinicalTrials.gov via https://clinicaltrials.gov/ and ISRCTN via https://www.isrctn.com/ for additional registered studies.

Study records

Search strategy

The search strategy will be guided by a medical librarian (NT) who will provide support and guidance on search terms and keywords. Broad search terms will be used to capture the maximum number of publications in this area. MeSH terms and word truncations will be used where appropriate. An example search strategy is outlined in Appendix 1. Once the initial searches have been performed

For peer review only - http://bmjopen.bmj.com/site/about/guidelines.xhtml

and a list of studies for inclusion has been agreed, we will conduct further searches if new relevant search terms are identified.

Data extraction and management

Identified references will be downloaded to reference library software for the initial title and abstract screening; this will be handled using Rayyan software [17]. All searches will be saved for referencing, including a full list of papers and timeline of searches. The processing of each paper will be clearly documented throughout the screening and review process.

Selection process

1- Once duplicates have been removed, two researchers will screen the titles and abstracts to exclude any evidently ineligible manuscripts using Rayyan software. Any disagreements will be included in the full-title review.
2- The full text of studies identified as potentially eligible following title and abstract screening will be assessed for inclusion by the two reviewers. Any disagreements in inclusion/exclusion of the paper will be reviewed and discussed with a third reviewer.
3- Once articles are selected, hand-searching will be used to review their references to look for relevant articles not already identified. This is of particular importance as healthcare use of ambulatory monitoring devices or non-published articles may be identified using this method. If any relevant search terms are identified these will be included in a new search strategy following the above process.
4- The included articles will then be assessed by the two reviewers. Authors will be contacted by email for clarification or for further data wherever necessary. We will use a pilot data extraction form. The extraction output will be compared and a final data extraction form will be created. Extraction will include the author data, country, type of publication, date of publication, registration details, study design, setting, numbers of patients, eligibility criteria, missing data, ambulatory monitoring systems under study and type of measurement (intermittent/continuous), standard care comparator and type of comparator (intermittent/continuous), vital signs and other metrics measured, primary outcomes, secondary outcomes and other relevant metrics, alerting system (if there was one and how clinical staff was alerted), effect sizes and other estimates, population, demographics and cohort data, main conclusions, quality assessment and risk of bias assessment.

An overview of the selection process may be found in Figure 1.

Quality assessment

Methodological quality of randomised controlled trials (RCTs) will be assessed using the Jadad Scale [18]. For risk of bias and internal validity assessment we will use the Cochrane risk of bias tool (RoB 2) [19] for randomised trials and the Newcastle Ottawa Scale for non-randomised studies [20] . Additionally, we will also use the Mixed Methods Appraisal tool (MMAT) [21].

For peer review only - http://bmjopen.bmj.com/site/about/guidelines.xhtml

Quality will be independently assessed and scores agreed by two reviewers; for significantly different scores and disagreements, a third reviewer will also conduct an independent quality assessment of the full-text. Scores will then be discussed and agreed between the 3 reviewers.

Where possible, visual assessment of funnel plots and Egger's regression will be used to assess publication bias. The GRADE (Grading of Recommendations Assessment, Development and Evaluation) methodology will be used across studies to report the overall strength of the review as high, moderate, low or very low [22].

Data Synthesis

Quantitative or narrative approach to synthesising data

We will classify studies according to the interventions which are assessed. If we identify at least three studies assessing the effect of relatively homogeneous interventions, we will perform meta-analyses to combine the results. These meta-analyses will be on the basis of the outcome measures mentioned above. Therefore, it is likely that the effect measures of interest will be risk ratios or hazard ratios. If similar outcomes measures are reported using different effect measures, or time horizons, we will convert to a universal scale where possible, using standard methods. Effect measures will be combined using a random-effects meta-analysis. We will report the combined result (with 95% confidence interval), a measure of between study heterogeneity, and the 95% prediction interval. Where appropriate we will use sensitivity analyses to look at the effect of including/excluding non-randomised studies in the meta-analyses.

Meta analyses will be graphically represented using forest plots. An assessment of heterogeneity will be made (using both the $\chi 2$ test and the I2 statistic) and estimates of effects will be extracted/calculated in accordance with the Cochrane Handbook for Systematic Reviews of Interventions version 6.0 [23]. Statistical analyses will be carried out using R software.

If there are too few homogeneous studies to enable meta-analysis, results will instead be described through narrative synthesis. In this case, we will also explore different hospital environments and standard monitoring impact on the reported outcomes (eg. medical ward using standard intermittent monitoring as comparator or ICU using continuous monitoring) and other relevant sub-group analysis as outlined below.[23][23][23](PT Higgins, Li, and K Deeks 2019)[23]

For peer review only

Subgroup analysis

If possible we will explore heterogeneity between studies, through sensitivity and subgroup analyses. This will be undertaken only in studies with acceptable statistical methods and appropriate outcome reporting. These include the following:

1. According to different standard care comparator (eg. intermittent/manual or continuous monitoring).
2. Ward environment (ICU/Surgical/Medical)
3. Stratification according to each vital sign
4. Stratification according to device used
5. Sub-group analysis of non-adult population

For peer review only - http://bmjopen.bmj.com/site/about/guidelines.xhtml

Clinical trial registries analysis

If any eligible studies are identified through searches of ClinicalTrialsGov and ISRCTN registries we will perform a narrative synthesis exploring the proportion of registered studies against results published (considering the reported end date).

Missing data

Where missing or incorrect data are identified, corresponding authors will be contacted to clarify.

Amendments to protocol

To ensure transparency, any change from this protocol will be documented and dated accordingly. No changes will be made to the main content of the protocol and any unexpected additional findings will be discussed in the systematic review.

Patient and public involvement

This systematic review is part of the vHDU project, aiming to implement ambulatory vital sign monitoring in the clinical environment. As this project develops, we will invite patients and members of the public to a group for ongoing support and feedback throughout the rest of the project. We have attended a number of local public engagement events, where members of the public showed interest and engaged in our vision of continuous wearable vital sign monitoring.

Author contributions

CA designed the study. CA, LY, SV and NT designed the search strategy. NT will conduct databases search. CA, LY, SV, CB, MP, MS and SG will be involved in data extraction and the assessment of methodological quality. PW and LT made final approval of the study protocol. CA wrote the initial draft of this paper and all authors contributed to and reviewed the final manuscript.

Data statement

Screening and data collection tables can be made available upon request.

Funding

The research was funded by the National Institute for Health Research (NIHR) Oxford Biomedical Research Centre (Award/Grant number: NIHR BRC-1215-20008). PW and LT are supported by the NIHR Biomedical Research Centre, Oxford. The views expressed are those of the author(s) and not necessarily those of the NHS, the NIHR or the Department of Health.

For peer review only - http://bmjopen.bmj.com/site/about/guidelines.xhtml

For peer review only

The funders have had no role in the study protocol design or the preparation of this manuscript, and will have no role in the collection, management, analysis and interpretation of the data or the writing of the final report.

Conflicts of interest

PW and LT report significant grants from the National Institute of Health Research (NIHR), UK and the NIHR Biomedical Research Centre, Oxford, during the conduct of the study. PW and LT report modest grants and personal fees from Sensyne Health, outside the submitted work. LT works part-time for Sensyne Health and has share options in the company. PW holds shares in the company.

Abbreviations

AMS – Ambulatory Monitoring System

ECG – Electrocardiogram

PPG - Photoplethysmography

EWS – Early Warning Scores

ICU – Intensive Care Unit

RCT – Randomised Controlled Trial

RRT – Rapid response team

References

1 NICE Clinical Guidelines. Acutely ill adults in hospital: recognising and responding to deterioration. 2007;:1–30.

2 Prgomet M, Cardona-Morrell M, Nicholson M, *et al.* Vital signs monitoring on general wards: clinical staff perceptions of current practices and the planned introduction of continuous monitoring technology. *Int J Qual Heal Care* 2016;**28**:515–21. doi:10.1093/intqhc/mzw062

3 Clifton DA, Clifton L, Sandu DM, *et al.* 'Errors' and omissions in paper-based early warning scores: The association with changes in vital signs-a database analysis. *BMJ Open* 2015;**5**:1–7. doi:10.1136/bmjopen-2014-007376

4 Jansen JO, Cuthbertson BH. Detecting critical illness outside the ICU: the role of track and trigger systems. *Curr Opin Crit Care* 2010;**16**:184–90. doi:10.1097/MCC.0b013e328338844e

5 Tarassenko L, Hann A, Young D. Integrated monitoring and analysis for early warning of patient deterioration. *Br J Anaesth* 2006;**97**:64–8. doi:10.1093/BJA/AEL113

6 Bonnici T, Tarassenko L, Clifton DA, *et al.* The digital patient. *Clin Med* 2013;**13**:252–7. doi:10.7861/clinmedicine.13-3-252

7 Downey CL, Chapman S, Randell R, *et al.* The impact of continuous versus intermittent vital signs monitoring in hospitals: A systematic review and narrative synthesis. *Int J Nurs Stud*

For peer review only - http://bmjopen.bmj.com/site/about/guidelines.xhtml

2018;**84**:19–27. doi:10.1016/J.IJNURSTU.2018.04.013

8 Javanbakht M, Mashayekhi A, Trevor M, *et al.* Cost utility analysis of continuous and intermittent versus intermittent vital signs monitoring in patients admitted to surgical wards. *J Med Econ* 2020;:1–9. doi:10.1080/13696998.2020.1747474

9 Appelboom G, Camacho E, Abraham ME, *et al.* Smart wearable body sensors for patient self-assessment and monitoring. *Arch Public Heal* 2014;**72**:28. doi:10.1186/2049-3258-72-28

10 Weenk M, van Goor H, Frietman B, *et al.* Continuous Monitoring of Vital Signs Using Wearable Devices on the General Ward: Pilot Study. *JMIR mHealth uHealth* 2017;**5**:e91. doi:10.2196/mhealth.7208

11 Louie A, Feiner JR, Bickler PE, *et al.* Four Types of Pulse Oximeters Accurately Detect Hypoxia during Low Perfusion and Motion. *Anesthesiology* 2018;**128**:520–30. doi:10.1097/ALN.0000000000002002

12 Sun L, Joshi M, Khan SN, *et al.* Clinical impact of multi-parameter continuous non-invasive monitoring in hospital wards: a systematic review and meta-analysis. *J R Soc Med* 2020;**113**:217–24. doi:10.1177/0141076820925436

13 Leenen JPL, Leerentveld C, van Dijk JD, *et al.* Current Evidence for Continuous Vital Signs Monitoring by Wearable Wireless Devices in Hospitalized Adults: Systematic Review. *J Med Internet Res* 2020;**22**:e18636. doi:10.2196/18636

14 Naziyok TP, Zeleke AA, Röhrig R. Contactless Patient Monitoring for General Wards: A Systematic Technology Review. *Stud Health Technol Inform* 2016;**228**:707–11.

15 Moher D, Liberati A, Tetzlaff J, *et al.* Preferred reporting items for systematic reviews and meta-analyses: the PRISMA statement. *PLoS Med* 2009;**6**:e1000097. doi:10.1371/journal.pmed.1000097

16 Moher D, Shamseer L, Clarke M, *et al.* Preferred reporting items for systematic review and meta-analysis protocols (PRISMA-P) 2015 statement. *Syst Rev* 2015;**4**:1. doi:10.1186/2046-4053-4-1

17 Ouzzani M, Hammady H, Fedorowicz Z, *et al.* Rayyan—a web and mobile app for systematic reviews. *Syst Rev* 2016;**5**:210. doi:10.1186/s13643-016-0384-4

18 Jadad AR, Moore RA, Carroll D, *et al.* Assessing the quality of reports of randomized clinical trials: is blinding necessary? *Control Clin Trials* 1996;**17**:1–12. doi:10.1016/0197-2456(95)00134-4

19 Sterne JAC, Savović J, Page MJ, *et al.* RoB 2: a revised tool for assessing risk of bias in randomised trials. *BMJ* 2019;**366**:l4898. doi:10.1136/bmj.l4898

20 Wells G, Shea B, O'Connell D, *et al.* The Newcastle-Ottawa Scale (NOS) for assessing the quality of nonrandomised studies in meta-analyses. Ottawa Hosp. Res. Inst.

21 Hong QN, Pluye P, Fàbregues S, *et al.* Mixed Methods Appraisal Tool (MMAT) Version 2018. McGIll Dep. Fam. Med. 2018.http://mixedmethodsappraisaltoolpublic.pbworks.com/w/file/fetch/127425851/MMAT_2018_criteria-manual_2018-04-04.pdf (accessed 15 Jul 2020).

22 Balshem H, Helfand M, Schünemann HJ, *et al.* GRADE guidelines: 3. Rating the quality of evidence. *J Clin Epidemiol* 2011;**64**:401–6. doi:10.1016/j.jclinepi.2010.07.015

23 PT Higgins J, Li T, K Deeks J. Chapter 6: Choosing effect measures and computing estimates of

For peer review only - http://bmjopen.bmj.com/site/about/guidelines.xhtml

effect. In: *Cochrane Handbook for Systematic Reviews of Interventions version 6.0*. 2019. www.training.cochrane.org/handbook

For peer review only

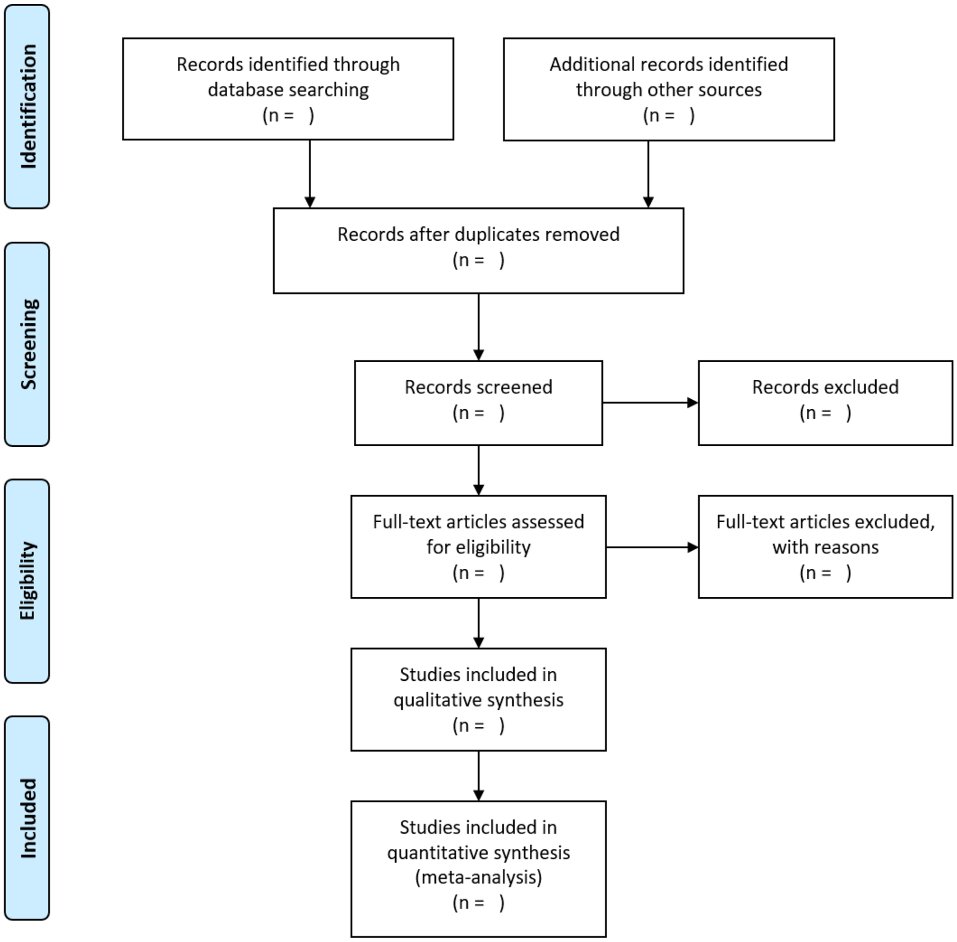

PRISMA Flowchart

Appendix 1 – Example Medline search terms

Database: Medline (Ovid MEDLINE® Epub Ahead of Print, In-Process & Other Non-Indexed Citations, Ovid MEDLINE® Daily and Ovid MEDLINE®) 1946 to present

Search Strategy: (09/07/20)

----

1 exp Monitoring, Ambulatory/ (28418)

2 ambulatory.tw. (77902)

3 exp Wearable Electronic Devices/ (11662)

4 wearable.tw. (11665)

5 (patch or patches).tw. (105886)

6 Adhesives/ (6649)

7 adhesive*.tw. (57238)

8 (sticker* or stick or sticks).tw. (12154)

9 (watch or watches).tw. (8921)

10 smartwatch*.tw. (294)

11 telemonitor*.tw. (1494)

12 tele monitor*.tw. (142)

13 exp Telemetry/ (13234)

14 telemetr*.tw. (8633)

15 clothing/ or shoes/ (15092)

16 cloth*.tw. (15965)

17 exp Textiles/ (6332)

18 textile*.tw. (10631)

19 exp Telemedicine/ (28526)

20 telemedicine*.tw. (10525)

21 exp Biosensing Techniques/ (54482)

22 biosens*.tw. (36571)

23 glove*.tw. (10538)

24 shoe*.tw. (9220)

For peer review only - http://bmjopen.bmj.com/site/about/guidelines.xhtml

25 accessor*.tw. (44010)

26 Wrist/ (8964)

27 wrist*.tw. (38228)

28 neckless*.tw. (22)

29 (belt or belts).tw. (12795)

30 armband*.tw. (577)

31 (shirt or shirts).tw. (1060)

32 contactless.tw. (1696)

33 cableless.tw. (8)

34 contact sensor*.tw. (179)

35 unobtrusive.tw. (1574)

36 remote.tw. (69057)

37 Wireless Technology/ (3552)

38 wireless*.tw. (14442)

39 non contact.tw. (5188)

40 noncontact.tw. (4678)

41 without wires.tw. (21)

42 non-restrict*.tw. (551)

43 unrestrict*.tw. (8498)

44 Automation/ (18073)

45 automat*.tw. (227620)

46 webcam*.tw. (392)

47 web-cam*.tw. (172)

48 video*.tw. (124969)

49 2 or 3 or 4 or 5 or 6 or 7 or 8 or 9 or 10 or 11 or 12 or 13 or 14 or 15 or 16 or 17 or 18 or 19 or 20 or 21 or 22 or 23 or 24 or 25 or 26 or 27 or 28 or 29 or 30 or 31 or 32 or 33 or 34 or 35 or 36 or 37 or 38 or 39 or 40 or 41 or 42 or 43 or 44 or 45 or 46 or 47 or 48 (967877)

50 Monitoring, Physiologic/mt [Methods] (18050)

51 monitor*.tw. (797034)

52 Oximetry/ (12956)

53 oximeter*.tw. (4028)

54 tracker*.tw. (4467)

For peer review only - http://bmjopen.bmj.com/site/about/guidelines.xhtml

55 Radar/ (1194)

56 radar*.tw. (5222)

57 Ballistocardiography/ (2047)

58 ballisto*.tw. (1828)

59 exp Accelerometry/ (8777)

60 accelerom*.tw. (16497)

61 gyro*.tw. (4713)

62 Photoplethysmography/ (1976)

63 ppg.tw. (3402)

64 photople*.tw. (3259)

65 videopleth*.tw. (4)

66 video pleth*.tw. (6)

67 photo pleth*.tw. (94)

68 Thermography/ (7557)

69 thermograph*.tw. (6084)

70 Infrared Rays/ (13185)

71 infrared*.tw. (135813)

72 thermal.tw. (197645)

73 thermistor*.tw. (1741)

74 camera*.tw. (45063)

75 wavelet analysis/ (2179)

76 wavelet*.tw. (11170)

77 waveform*.tw. (28312)

78 ambient light*.tw. (1869)

79 piezoelectric*.tw. (10331)

80 piezo electric*.tw. (342)

81 Electric Impedance/ (17049)

82 impedan*.tw. (44627)

83 exp Electrocardiography/ (203872)

84 electrocar*.tw. (87880)

85 (ecg or ecgs).tw. (65216)

For peer review only

86 (ekg or ekgs).tw. (3072)

87 electrom*.tw. (96809)

88 electrog*.tw. (20210)

89 exp Electrodes/ (129544)

90 electrode*.tw. (159855)

91 50 or 51 or 52 or 53 or 54 or 55 or 56 or 57 or 58 or 59 or 60 or 61 or 62 or 63 or 64 or 65 or 66 or 67 or 68 or 69 or 70 or 71 or 72 or 73 or 74 or 75 or 76 or 77 or 78 or 79 or 80 or 81 or 82 or 83 or 84 or 85 or 86 or 87 or 88 or 89 or 90 (1778658)

92 49 and 91 (174705)

93 1 or 92 (191231)

94 vital signs/ or blood pressure/ or body temperature/ or heart rate/ or respiratory rate/ (409195)

95 heart rate*.tw. (158333)

96 pulse rate*.tw. (7569)

97 pulse*.tw. (254455)

98 respiratory rate*.tw. (14849)

99 breathing rate*.tw. (1219)

100 respiration.tw. (66550)

101 breathing.tw. (70922)

102 blood pressure.tw. (292754)

103 systolic.tw. (167582)

104 diastolic.tw. (124099)

105 perfusion.tw. (159241)

106 spo2.tw. (5003)

107 oxygenation.tw. (51448)

108 oxygen saturation.tw. (25872)

109 temperature*.tw. (644652)

110 exp Fever/ (43219)

111 fever*.tw. (170228)

112 hypertherm*.tw. (35091)

113 pyrex*.tw. (5318)

114 Hypothermia/ (13882)

For peer review only - http://bmjopen.bmj.com/site/about/guidelines.xhtml

For peer review only

115 hypotherm*.tw. (41774)

116 apyrex*.tw. (209)

117 a-pyrex*.tw. (173)

118 vital sign*.tw. (14684)

119 vitals.tw. (484)

120 vital param*.tw. (997)

121 vital function*.tw. (2764)

122 94 or 95 or 96 or 97 or 98 or 99 or 100 or 101 or 102 or 103 or 104 or 105 or 106 or 107 or 108 or 109 or 110 or 111 or 112 or 113 or 114 or 115 or 116 or 117 or 118 or 119 or 120 or 121 (2024979)

123 Hospitalization/ (107045)

124 hospital*.tw. (1274951)

125 inhospital.tw. (1742)

126 in-hospital.tw. (87915)

127 admitted.tw. (203836)

128 inpatient*.tw. (107148)

129 surgical.tw. (960793)

130 pacu.tw. (2474)

131 post anaesthesia care unit*.tw. (321)

132 post anesthesia care unit*.tw. (723)

133 postanaesthesia care unit*.tw. (190)

134 postanesthesia care unit*.tw. (1885)

135 icu.tw. (55877)

136 exp Critical Care/ (57601)

137 intensive care.tw. (142201)

138 itu.tw. (864)

139 aicu.tw. (45)

140 icus.tw. (10220)

141 itus.tw. (43)

142 aicus.tw. (7)

143 pacus.tw. (81)

144 critical care.tw. (27368)

For peer review only - http://bmjopen.bmj.com/site/about/guidelines.xhtml

145 (ward or wards).tw. (58951)

146 Triage/ (11643)

147 triage*.tw. (18408)

148 Emergencies/ (40252)

149 (emergency or emergencies).tw. (267369)

150 123 or 124 or 125 or 126 or 127 or 128 or 129 or 130 or 131 or 132 or 133 or 134 or 135 or 136 or 137 or 138 or 139 or 140 or 141 or 142 or 143 or 144 or 145 or 146 or 147 or 148 or 149 (2517115)

151 93 and 122 and 150 (5115)

For peer review only

For peer review only - http://bmjopen.bmj.com/site/about/guidelines.xhtml

PRISMA-P (Preferred Reporting Items for Systematic review and Meta-Analysis Protocols) 2015 checklist: recommended items to address in a systematic review protocol*

Section and topic	Item No	Checklist item	Page
**ADMINISTRATIVE INFORMATION**			
Title:			
Identification	1a	Identify the report as a protocol of a systematic review	1
Update	1b	If the protocol is for an update of a previous systematic review, identify as such	N/A
Registration	2	If registered, provide the name of the registry (such as PROSPERO) and registration number	2
Authors:			
Contact	3a	Provide name, institutional affiliation, e-mail address of all protocol authors; provide physical mailing address of corresponding author	1
Contributions	3b	Describe contributions of protocol authors and identify the guarantor of the review	11
Amendments	4	If the protocol represents an amendment of a previously completed or published protocol, identify as such and list changes; otherwise, state plan for documenting important protocol amendments	11
Support:			
Sources	5a	Indicate sources of financial or other support for the review	11
Sponsor	5b	Provide name for the review funder and/or sponsor	11
Role of sponsor or funder	5c	Describe roles of funder(s), sponsor(s), and/or institution(s), if any, in developing the protocol	11
**INTRODUCTION**			
Rationale	6	Describe the rationale for the review in the context of what is already known	3
Objectives	7	Provide an explicit statement of the question(s) the review will address with reference to participants, interventions, comparators, and outcomes (PICO)	4
**METHODS**			
Eligibility criteria	8	Specify the study characteristics (such as PICO, study design, setting, time frame) and report characteristics (such as years considered, language, publication status) to be used as criteria for eligibility for the review	4-6
Information sources	9	Describe all intended information sources (such as electronic databases, contact with study authors, trial registers or other grey literature sources) with planned dates of coverage	6
Search strategy	10	Present draft of search strategy to be used for at least one electronic database, including planned limits, such that it could be repeated	Appendix 1

For peer review only

Study records:			
Data management	11a	Describe the mechanism(s) that will be used to manage records and data throughout the review	7
Selection process	11b	State the process that will be used for selecting studies (such as two independent reviewers) through each phase of the review (that is, screening, eligibility and inclusion in meta-analysis)	7
Data collection process	11c	Describe planned method of extracting data from reports (such as piloting forms, done independently, in duplicate), any processes for obtaining and confirming data from investigators	7
Data items	12	List and define all variables for which data will be sought (such as PICO items, funding sources), any pre-planned data assumptions and simplifications	7
Outcomes and prioritization	13	List and define all outcomes for which data will be sought, including prioritization of main and additional outcomes, with rationale	5-6
Risk of bias in individual studies	14	Describe anticipated methods for assessing risk of bias of individual studies, including whether this will be done at the outcome or study level, or both; state how this information will be used in data synthesis	8-9
Data synthesis	15a	Describe criteria under which study data will be quantitatively synthesised	9
	15b	If data are appropriate for quantitative synthesis, describe planned summary measures, methods of handling data and methods of combining data from studies, including any planned exploration of consistency (such as I^2, Kendall's τ)	9
	15c	Describe any proposed additional analyses (such as sensitivity or subgroup analyses, meta-regression)	9
	15d	If quantitative synthesis is not appropriate, describe the type of summary planned	9
Meta-bias(es)	16	Specify any planned assessment of meta-bias(es) (such as publication bias across studies, selective reporting within studies)	9
Confidence in cumulative evidence	17	Describe how the strength of the body of evidence will be assessed (such as GRADE)	9

*** It is strongly recommended that this checklist be read in conjunction with the PRISMA-P Explanation and Elaboration (cite when available) for important clarification on the items. Amendments to a review protocol should be tracked and dated. The copyright for PRISMA-P (including checklist) is held by the PRISMA-P Group and is distributed under a Creative Commons Attribution Licence 4.0.**

From: Shamseer L, Moher D, Clarke M, Ghersi D, Liberati A, Petticrew M, Shekelle P, Stewart L, PRISMA-P Group. Preferred reporting items for systematic review and meta-analysis protocols (PRISMA-P) 2015: elaboration and explanation. BMJ. 2015 Jan 2;349(jan02 1):g7647.

For peer review only

BMJ Open

Protocol for a systematic review assessing ambulatory vital sign monitoring impact on deterioration detection and related clinical outcomes in hospitalised patients.

Journal:	*BMJ Open*
Manuscript ID	bmjopen-2020-047715.R1
Article Type:	Protocol
Date Submitted by the Author:	30-Mar-2021
Complete List of Authors:	Areia, Carlos; University of Oxford, Nuffield Department of Clinical Neurosciences<br>Vollam, Sarah; University of Oxford, Nuffield Department of Clinical Neurosciences<br>Young, Louise; University of Oxford, Nuffield Department of Clinical Neurosciences<br>Biggs, Christopher; University of Oxford, Nuffield Department of Clinical Neurosciences<br>Pimentel, Marco; University of Oxford, Institute of Biomedical Engineering, Department of Engineering Science<br>Santos, Mauro; University of Oxford, Institute of Biomedical Engineering, Department of Engineering Science<br>Thurley, Neal; University of Oxford, Bodleian Health Care Libraries<br>Gerry, Stephen; University of Oxford, Centre for Statistics in Medicine<br>Tarassenko, Lionel; University of Oxford, Institute of Biomedical Engineering, Department of Engineering Science<br>Watkinson, Peter; University of Oxford, Nuffield Department of Clinical Neurosciences
**Primary Subject Heading**:	Health informatics
Secondary Subject Heading:	Nursing
Keywords:	Health informatics < BIOTECHNOLOGY & BIOINFORMATICS, Telemedicine < BIOTECHNOLOGY & BIOINFORMATICS, ANAESTHETICS, BIOTECHNOLOGY & BIOINFORMATICS

SCHOLARONE™
Manuscripts

I, the Submitting Author has the right to grant and does grant on behalf of all authors of the Work (as defined in the below author licence), an exclusive licence and/or a non-exclusive licence for contributions from authors who are: i) UK Crown employees; ii) where BMJ has agreed a CC-BY licence shall apply, and/or iii) in accordance with the terms applicable for US Federal Government officers or employees acting as part of their official duties; on a worldwide, perpetual, irrevocable, royalty-free basis to BMJ Publishing Group Ltd ("BMJ") its licensees and where the relevant Journal is co-owned by BMJ to the co-owners of the Journal, to publish the Work in this journal and any other BMJ products and to exploit all rights, as set out in our licence.

The Submitting Author accepts and understands that any supply made under these terms is made by BMJ to the Submitting Author unless you are acting as an employee on behalf of your employer or a postgraduate student of an affiliated institution which is paying any applicable article publishing charge ("APC") for Open Access articles. Where the Submitting Author wishes to make the Work available on an Open Access basis (and intends to pay the relevant APC), the terms of reuse of such Open Access shall be governed by a Creative Commons licence – details of these licences and which Creative Commons licence will apply to this Work are set out in our licence referred to above.

Other than as permitted in any relevant BMJ Author's Self Archiving Policies, I confirm this Work has not been accepted for publication elsewhere, is not being considered for publication elsewhere and does not duplicate material already published. I confirm all authors consent to publication of this Work and authorise the granting of this licence.

For peer review only

Title: Protocol for a systematic review assessing ambulatory vital sign monitoring impact on deterioration detection and related clinical outcomes in hospitalised patients.

Authors:

Carlos Areia[1]

Sarah Vollam[1]

Louise Young[1]

Christopher Biggs[1]

Marco Pimentel[2]

Mauro Santos[2]

Neal Thurley[3]

Stephen Gerry [4]

Lionel Tarassenko[2]

Peter Watkinson[1]

[1] Critical Care Research Group, Nuffield Department of Clinical Neurosciences, University of Oxford, Oxford, Oxfordshire, UK

[2] Institute of Biomedical Engineering, Department of Engineering Science, University of Oxford, Oxford, Oxfordshire, UK

[3] Bodleian Health Care Libraries, University of Oxford, Oxford, Oxfordshire, UK

[4] Centre for Statistics in Medicine, University of Oxford, Oxford, Oxfordshire, UK

Corresponding author:

Carlos Areia

Critical Care Research Group, Nuffield Department of Clinical Neurosciences, University of Oxford

Kadoorie Research Centre, John Radcliffe Hospital, Oxford, Oxfordshire, UK

OX3 9DU

carlos.morgadoareia@ndcn.ox.ac.uk

For peer review only - http://bmjopen.bmj.com/site/about/guidelines.xhtml

Word count - 3300

For peer review only - http://bmjopen.bmj.com/site/about/guidelines.xhtml

Abstract

Introduction

Ambulatory monitoring systems (AMS) can facilitate early detection of clinical deterioration, and have the potential to improve hospitalised patient outcomes. The objective of this systematic review is to assess the impact of vital signs monitoring on detection of deterioration and related outcomes in hospitalised patients using ambulatory monitoring systems, in comparison with standard care.

Methods and analysis

A systematic search was conducted on 27th August 2020 in MEDLINE, Embase, CINAHL, Cochrane Database of Systematic Reviews, CENTRAL and Health Technology Assessment databases, as well as grey literature. Search results will be reviewed in accordance with the Preferred Reporting Items for Systematic Review and Meta-Analysis checklist for systematic reviews. Studies comparing the use of ambulatory monitoring devices against standard care for deterioration detection and related clinical outcomes in hospitalised patients will be included and further clinical and other outcomes will also be explored. Deterioration related outcomes may include (but not limited to) unplanned intensive care admissions, rapid response team activation and unscheduled emergency interventions, as defined by the included studies. Two reviewers will independently extract study data and assess the quality and risk of bias of included studies. Where possible, a meta-analysis will be conducted and quantitative results presented. Alternatively, a narrative synthesis will be reported.

Ethics and Dissemination

Ethical approval is not required for this study as no primary data will be collected. This study is part of our virtual High Dependency Unit (vHDU) project and will be disseminated through peer-reviewed publications, public and scientific conference presentations.

PROSPERO Registration number

CRD42020188633

Keywords

ambulatory monitoring; deterioration detection; wearables; vital signs;

Strength and limitations of the study

- A potential limitation of this review is the focus on ambulatory monitoring systems that may limit the final number of included studies.
- This is the first systematic review assessing the effect of ambulatory monitoring devices on deterioration detection and related clinical outcomes in comparison with standard care.
- This review may provide an indication of the impact of ambulatory monitoring devices use in the hospital environment.
- We will review clinical trial registries and report the proportion of published studies against registered.

For peer review only - http://bmjopen.bmj.com/site/about/guidelines.xhtml

For peer review only - http://bmjopen.bmj.com/site/about/guidelines.xhtml

Background

The utilisation of physiological early warning scoring (EWS) systems which monitor “standard” vital signs, including pulse rate, respiratory rate, blood pressure, oxygen saturations and temperature) remains current practice, coupled with a graded response such as referral for a senior review or increasing monitoring frequency [1]. This frequency of observations is generally guided by the clinical condition of the patient. However, intermittent measurement of vital signs (including application of devices and documentation) can be time consuming for healthcare professionals [2] and therefore, the desired monitoring frequency of is often not achieved [3]. This is identified as a limitation of intermittent monitoring systems, as they are dependent on the frequency of physiological monitoring observations [4]; of more concern is that even when ideal frequency is achieved, patients might deteriorate between observation sets [5].

Higher risk patients are often continuously monitored, improving early detection of deterioration [2]. However, in the UK, continuous monitoring is not commonly used in the ward environment [6]. In a systematic review, Downey and colleagues (2018) suggested that continuous vital sign monitoring outside the critical care setting may be feasible and has the potential to improve patient outcomes when compared to intermittent monitoring [7,8]. Another study suggested that implementing continuous monitoring in surgical wards was also cost effective [8]. On the other hand, clinical staff interviews indicate that despite the benefits and potential to increase the timely detection of patient deterioration, limitations in continuous vital sign monitoring technology can pose a barrier to implementation [2]. One of the main suggested reasons identified was invasiveness and restricted mobility [6,7].

As a response to current limitations in healthcare monitoring, companies are extending the capabilities of commercially available wearable ambulatory vital sign monitoring systems [9]. These non-wired monitors may provide an alternative continuous monitoring system, affording patients more mobility, less discomfort, reduce nursing time and improve the early detection of abnormal physiological parameters [10]. Nevertheless, a major barrier to the clinical implementation of these systems is their unproven reliability, efficiency and data fidelity [9]. Another challenge is the potential detrimental effect of motion on the data derived from such monitoring systems, for example, motion is known to affect the accuracy of pulse oximetry readings [11]. This was seen throughout a clinically relevant range of measurements, with less accuracy at lower arterial oxygen saturations, which is clearly highly undesirable in clinical practice.

A recent systematic review and meta-analysis assessed the impact of multi-parameter continuous non-invasive monitoring in hospital wards, suggesting that patients monitored in this way had a 39% decreased mortality risk when compared to those receiving intermittent monitoring; as well as a trend of reduced intensive care unit (ICU) transfer, rapid response team activation and hospital length of stay [12]. However, this review focused on non-invasive continuous monitoring and excluded neonatal, paediatric, obstetric, ICU and high-risk acute patients. Another recent review analysed the validation, feasibility, clinical outcomes and costs of 13 different wearable devices and concluded that these were predominantly in the validation and feasibility testing phases [13], highlighting the lack of studies exploring clinical outcomes. However, this review focused on recent wearable devices, and only including studies between 2009-2019. Furthermore, while there are many devices claiming the ability to safely monitor patients at risk of deterioration [14], evidence assessing the impact of ambulatory monitoring systems in the ward environment remains inconclusive, limiting implementation and clinical use [14].

For peer review only - http://bmjopen.bmj.com/site/about/guidelines.xhtml

Aims and objectives

The objective of this systematic review is to assess the impact of vital sign monitoring in the deterioration detection and related clinical outcomes of hospitalised patients using ambulatory monitoring systems in comparison with standard care.

Methods and Analysis

This systematic review was registered with the International Prospective Register of Systematic Reviews (PROSPERO) on the 10th July 2020 (registration number CRD42020188633). This review will be conducted in accordance with the Preferred Reporting Items for Systematic Reviews and Meta-Analyses (PRISMA) checklist [15]. This protocol is in accordance with the PRISMA-Protocol (PRISMA-P) statement checklist [16].

Methodological considerations

Inclusion and exclusion criteria

Types of studies

Randomised controlled trials, interventional studies, observational studies (including case-control or cohort studies) and pilot studies will be included. Retrospective studies that comply with the proposed outcomes and eligibility criteria will be considered. To minimise publication bias, unpublished studies will also be included. There will be no date or language restrictions as every effort will be attempted to translate a non-English article.

Systematic, narrative and scoping reviews will be excluded, but we may use relevant reviews to facilitate identification of original data where appropriate. Diagnostic accuracy and validation studies purely assessing device accuracy or reliability will also be excluded. However, where possible for the included studies, we will extract appropriate diagnostic accuracy metrics as a secondary outcome.

Types of participants

We will include any studies conducted in the hospital environment. Although we are only including data from admitted patients, there are no restrictions in minimum length of stay or monitoring period. Studies conducted in healthy volunteers or non-hospitalised patients will be excluded.

Interventions

Studies that include non- or minimally invasive ambulatory systems not part of standard care will be included. As per it's definition, ambulatory monitoring systems (AMS) should not be restrictive or pose a barrier to patient mobility. To be included, AMS should monitor one or more vital sign (heart rate, respiratory rate, temperature, blood pressure or oxygen saturation) either continuously or intermittently (eg. sending streams of data every 5 minutes) and measurements should be automated, therefore requiring frequent manual input from the clinical staff. These systems can additionally:

- include one or more ambulatory monitoring devices;

For peer review only - http://bmjopen.bmj.com/site/about/guidelines.xhtml

- provide any other measurements that might be considered for analysis (such as ECG, PPG, accelerometry, etc…) as long as the device measures at least one vital sign.

Comparators

For our primary outcome we will include studies that compare the ambulatory monitoring systems to standard care. Standard ward care can include (but is not limited to) the following measurements:

- **Heart rate:** Manual pulse rate count, spot checks using blood pressure machine/pulse oximeter, derived from PPG, ECG or arterial pressure waveforms.
- **Respiratory rate:** Manual breathing rate count, derived from capnography or ECG waveform.
- **Blood pressure:** Blood pressure cuff reading, derived from arterial pressure waveform.
- **Oxygen saturation:** Pulse oximetry, arterial blood gas.
- **Temperature:** oropharyngeal, nasopharyngeal, rectal and skin thermometers.

If possible, we will describe the frequency of intermittent observations in studies using manual vital sign measurements as standard care.

If enough studies are available, we will do a sub-group analysis according to the type of standard monitoring (intermittent manual vital sign measurement or automated measurements) as these may vary between studies and wards (eg. intensive care units using standard continuous wired monitoring).

As a secondary outcome, we will also include studies comparing different devices, if they comply with the eligibility criteria.

Outcomes

We will include studies measuring deterioration detection and related clinical outcomes. If relevant, we will also include studies reporting other clinical outcomes in hospitalised patients similar to other reviews [12]. It is likely there may be a great variety of outcomes measurements not only on patient outcomes but also in devices reliability and efficacy. Depending on the quantity and quality of papers fulfilling eligibility criteria and being included in the review; we may report a qualitative synthesis instead of a quantitative analysis.

Primary Outcomes

We will aim to compare the ambulatory monitoring systems with standard care in regard to deterioration detection and related clinical outcomes metrics. We will be flexible, as we predict studies may use different outcomes and time frames. These can include (but are not limited to):

1- ICU admission
2- Rapid response team (RRT) activation
3- Complications and adverse events
4- Emergency/unscheduled interventions (eg. cardio pulmonary resuscitation, surgery, antibiotics, etc…)

For peer review only

For peer review only - http://bmjopen.bmj.com/site/about/guidelines.xhtml

Secondary Outcomes

As a secondary outcome, we will report any other relevant patient related outcomes (eg. mortality, length of stay and acute changes in therapy/medication). Similarly, these metrics are likely to vary between studies so there will be no pre-determined criteria for inclusion at this point.

If enough data are available, we will also report device accuracy and reliability measurements, as well as the level of agreement between ambulatory monitoring systems and the standard care comparator (where available). We will also narratively explore patient experience and satisfaction with the AMS in the included studies.

Exploratory Outcomes

We will narratively report alerting systems used and explore their impact on clinical outcomes for the included studies, where reported. This may include type of early warning score, alert thresholds used for each vital sign, false alert rates and other relevant alert parameters or information.

We will also perform a clinical trial registry search and narratively report the proportion and characteristics of eligible registered studies against the number of results published (linking the respective registered studies end date and timelines with published reports).

Information sources

Electronic searches

Relevant articles up to 27th August 2020 were identified through electronic searches MEDLINE Ovid (including Epub Ahead of Print and In-Process & Other Non-Indexed Citations), Embase (Ovid), CINAHL (EBSCO) and Cochrane Database of Systematic Reviews (Cochrane Library, Wiley), Cochrane Central Register of Controlled Trials (CENTRAL) (Cochrane Library, Wiley) and Health Technology Assessment (HTA) database via https://www.crd.york.ac.uk/CRDWeb/.

OpenGrey http://www.opengrey.eu/ was also searched for any unpublished grey literature. Given the popular culture/commercial applications of the technology, Google Scholar via https://scholar.google.com/ will also be searched. Additionally, we will also seek to identify eligible pre-prints using medRxiv via https://www.medrxiv.org/ . If relevant papers are identified through included studies references, these may also be included.

Finally, we also searched trial registries such as ClinicalTrials.gov via https://clinicaltrials.gov/ and ISRCTN via https://www.isrctn.com/ for additional registered studies.

Study records

Search strategy

The search strategy will be guided by a medical librarian (NT) who will provide support and guidance on search terms and keywords. Broad search terms will be used to capture the maximum number of publications in this area. MeSH terms and word truncations will be used where appropriate. An example search strategy is outlined in Appendix 1. Once the initial searches have been performed

For peer review only - http://bmjopen.bmj.com/site/about/guidelines.xhtml

and a list of studies for inclusion has been agreed, we will conduct further searches if new relevant search terms are identified.

Data extraction and management

Identified references will be downloaded to reference library software for the initial title and abstract screening; this will be handled using Rayyan software [17]. All searches will be saved for referencing, including a full list of papers and timeline of searches. The processing of each paper will be clearly documented throughout the screening and review process.

Selection process

1- Once duplicates have been removed, two researchers will screen the titles and abstracts to exclude any evidently ineligible manuscripts using Rayyan software. Any disagreements will be included in the full-title review.
2- The full text of studies identified as potentially eligible following title and abstract screening will be assessed for inclusion by the two reviewers. Any disagreements in inclusion/exclusion of the paper will be reviewed and discussed with a third reviewer.
3- Once articles are selected, hand-searching will be used to review their references to look for relevant articles not already identified. This is of particular importance as healthcare use of ambulatory monitoring devices or non-published articles may be identified using this method. If any relevant search terms are identified these will be included in a new search strategy following the above process.
4- The included articles will then be assessed by the two reviewers. Authors will be contacted by email for clarification or for further data wherever necessary. We will use a pilot data extraction form. The extraction output will be compared and a final data extraction form will be created. Extraction will include the author data, country, type of publication, date of publication, registration details, study design, setting, numbers of patients, eligibility criteria, missing data, ambulatory monitoring systems under study and type of measurement (intermittent/continuous), standard care comparator and type of comparator (intermittent/continuous), vital signs and other metrics measured, primary outcomes, secondary outcomes and other relevant metrics, alerting system (if there was one and how clinical staff was alerted), effect sizes and other estimates, population, demographics and cohort data, main conclusions, quality assessment and risk of bias assessment.

An overview of the selection process may be found in Figure 1.

Quality assessment

Methodological quality of randomised controlled trials (RCTs) will be assessed using the Jadad Scale [18]. For risk of bias and internal validity assessment we will use the Cochrane risk of bias tool (RoB 2) [19] for randomised trials and the Newcastle Ottawa Scale for non-randomised studies [20] . Additionally, we will also use the Mixed Methods Appraisal tool (MMAT) [21].

For peer review only

Quality will be independently assessed and scores agreed by two reviewers; for significantly different scores and disagreements, a third reviewer will also conduct an independent quality assessment of the full-text. Scores will then be discussed and agreed between the 3 reviewers.

Where possible, visual assessment of funnel plots and Egger's regression will be used to assess publication bias. The GRADE (Grading of Recommendations Assessment, Development and Evaluation) methodology will be used across studies to report the overall strength of the review as high, moderate, low or very low [22].

Data Synthesis

Quantitative or narrative approach to synthesising data

We will classify studies according to the interventions which are assessed. If we identify at least three studies assessing the effect of relatively homogeneous interventions, we will perform meta-analyses to combine the results. These meta-analyses will be on the basis of the outcome measures mentioned above. Therefore, it is likely that the effect measures of interest will be risk ratios or hazard ratios. If similar outcomes measures are reported using different effect measures, or time horizons, we will convert to a universal scale where possible, using standard methods. Effect measures will be combined using a random-effects meta-analysis. We will report the combined result (with 95% confidence interval), a measure of between study heterogeneity, and the 95% prediction interval. Where appropriate we will use sensitivity analyses to look at the effect of including/excluding non-randomised studies in the meta-analyses.

Meta analyses will be graphically represented using forest plots. An assessment of heterogeneity will be made (using both the $\chi 2$ test and the I2 statistic) and estimates of effects will be extracted/calculated in accordance with the Cochrane Handbook for Systematic Reviews of Interventions version 6.0 [23]. Statistical analyses will be carried out using R software.

If there are too few homogeneous studies to enable meta-analysis, results will instead be described through narrative synthesis. In this case, we will also explore different hospital environments and standard monitoring impact on the reported outcomes (eg. medical ward using standard intermittent monitoring as comparator or ICU using continuous monitoring) and other relevant sub-group analysis as outlined below.

Subgroup analysis

If possible we will explore heterogeneity between studies, through sensitivity and subgroup analyses. This will be undertaken only in studies with acceptable statistical methods and appropriate outcome reporting. These include the following:

1. According to different standard care comparator (eg. intermittent/manual or continuous monitoring).
2. Ward environment (ICU/Surgical/Medical)
3. Stratification according to each vital sign
4. Stratification according to device used
5. Sub-group analysis of non-adult population

For peer review only - http://bmjopen.bmj.com/site/about/guidelines.xhtml
For peer review only

Clinical trial registries analysis

If any eligible studies are identified through searches of ClinicalTrialsGov and ISRCTN registries we will perform a narrative synthesis exploring the proportion of registered studies against results published (considering the reported end date).

Missing data

Where missing or incorrect data are identified, corresponding authors will be contacted to clarify.

For peer review only

Amendments to protocol

To ensure transparency, any change from this protocol will be documented and dated accordingly. No changes will be made to the main content of the protocol and any unexpected additional findings will be discussed in the systematic review.

Patient and public involvement

This systematic review is part of the vHDU project, aiming to implement ambulatory vital sign monitoring in the clinical environment. As this project develops, we will invite patients and members of the public to a group for ongoing support and feedback throughout the rest of the project. We have attended a number of local public engagement events, where members of the public showed interest and engaged in our vision of continuous wearable vital sign monitoring.

Author contributions

CA designed the study. CA, LY, SV and NT designed the search strategy. NT will conduct databases search. CA, LY, SV, CB, MP, MS and SG will be involved in data extraction and the assessment of methodological quality. PW and LT made final approval of the study protocol. CA wrote the initial draft of this paper and all authors contributed to and reviewed the final manuscript.

Data statement

Screening and data collection tables can be made available upon request.

Funding

The research was funded by the National Institute for Health Research (NIHR) Oxford Biomedical Research Centre (Award/Grant number: NIHR BRC-1215-20008). PW and LT are supported by the NIHR Biomedical Research Centre, Oxford. The views expressed are those of the author(s) and not necessarily those of the NHS, the NIHR or the Department of Health.

The funders have had no role in the study protocol design or the preparation of this manuscript, and will have no role in the collection, management, analysis and interpretation of the data or the writing of the final report.

Conflicts of interest

PW and LT report significant grants from the National Institute of Health Research (NIHR), UK and the NIHR Biomedical Research Centre, Oxford, during the conduct of the study. PW and LT report modest grants and personal fees from Sensyne Health, outside the submitted work. LT works part-time for Sensyne Health and has share options in the company. PW holds shares in the company.

Ethics and Dissemination

Ethical approval is not required for this study as no primary data will be collected. Where appropriate, data access approvals might be sought for individual study data upon contact with the relevant author/research team. This study is part of our virtual High Dependency Unit (vHDU) project and will be disseminated through peer-reviewed publications, public and scientific conference presentations.

Abbreviations

AMS – Ambulatory Monitoring System

ECG – Electrocardiogram

PPG - Photoplethysmography

EWS – Early Warning Scores

ICU – Intensive Care Unit

RCT – Randomised Controlled Trial

RRT – Rapid response team

Figures

Figure 1 – PRISMA flow diagram

References

1 NICE Clinical Guidelines. Acutely ill adults in hospital: recognising and responding to deterioration. 2007;:1–30.

2 Prgomet M, Cardona-Morrell M, Nicholson M, *et al.* Vital signs monitoring on general wards: clinical staff perceptions of current practices and the planned introduction of continuous monitoring technology. *Int J Qual Heal Care* 2016;**28**:515–21. doi:10.1093/intqhc/mzw062

3 Clifton DA, Clifton L, Sandu DM, *et al.* 'Errors' and omissions in paper-based early warning scores: The association with changes in vital signs-a database analysis. *BMJ Open* 2015;**5**:1–7.

For peer review only - http://bmjopen.bmj.com/site/about/guidelines.xhtml

doi:10.1136/bmjopen-2014-007376

4 Jansen JO, Cuthbertson BH. Detecting critical illness outside the ICU: the role of track and trigger systems. *Curr Opin Crit Care* 2010;**16**:184–90. doi:10.1097/MCC.0b013e328338844e

5 Tarassenko L, Hann A, Young D. Integrated monitoring and analysis for early warning of patient deterioration. *Br J Anaesth* 2006;**97**:64–8. doi:10.1093/BJA/AEL113

6 Bonnici T, Tarassenko L, Clifton DA, *et al.* The digital patient. *Clin Med* 2013;**13**:252–7. doi:10.7861/clinmedicine.13-3-252

7 Downey CL, Chapman S, Randell R, *et al.* The impact of continuous versus intermittent vital signs monitoring in hospitals: A systematic review and narrative synthesis. *Int J Nurs Stud* 2018;**84**:19–27. doi:10.1016/J.IJNURSTU.2018.04.013

8 Javanbakht M, Mashayekhi A, Trevor M, *et al.* Cost utility analysis of continuous and intermittent versus intermittent vital signs monitoring in patients admitted to surgical wards. *J Med Econ* 2020;:1–9. doi:10.1080/13696998.2020.1747474

9 Appelboom G, Camacho E, Abraham ME, *et al.* Smart wearable body sensors for patient self-assessment and monitoring. *Arch Public Heal* 2014;**72**:28. doi:10.1186/2049-3258-72-28

10 Weenk M, van Goor H, Frietman B, *et al.* Continuous Monitoring of Vital Signs Using Wearable Devices on the General Ward: Pilot Study. *JMIR mHealth uHealth* 2017;**5**:e91. doi:10.2196/mhealth.7208

11 Louie A, Feiner JR, Bickler PE, *et al.* Four Types of Pulse Oximeters Accurately Detect Hypoxia during Low Perfusion and Motion. *Anesthesiology* 2018;**128**:520–30. doi:10.1097/ALN.0000000000002002

12 Sun L, Joshi M, Khan SN, *et al.* Clinical impact of multi-parameter continuous non-invasive monitoring in hospital wards: a systematic review and meta-analysis. *J R Soc Med* 2020;**113**:217–24. doi:10.1177/0141076820925436

13 Leenen JPL, Leerentveld C, van Dijk JD, *et al.* Current Evidence for Continuous Vital Signs Monitoring by Wearable Wireless Devices in Hospitalized Adults: Systematic Review. *J Med Internet Res* 2020;**22**:e18636. doi:10.2196/18636

14 Naziyok TP, Zeleke AA, Röhrig R. Contactless Patient Monitoring for General Wards: A Systematic Technology Review. *Stud Health Technol Inform* 2016;**228**:707–11.

15 Moher D, Liberati A, Tetzlaff J, *et al.* Preferred reporting items for systematic reviews and meta-analyses: the PRISMA statement. *PLoS Med* 2009;**6**:e1000097. doi:10.1371/journal.pmed.1000097

16 Moher D, Shamseer L, Clarke M, *et al.* Preferred reporting items for systematic review and meta-analysis protocols (PRISMA-P) 2015 statement. *Syst Rev* 2015;**4**:1. doi:10.1186/2046-4053-4-1

17 Ouzzani M, Hammady H, Fedorowicz Z, *et al.* Rayyan—a web and mobile app for systematic reviews. *Syst Rev* 2016;**5**:210. doi:10.1186/s13643-016-0384-4

18 Jadad AR, Moore RA, Carroll D, *et al.* Assessing the quality of reports of randomized clinical trials: is blinding necessary? *Control Clin Trials* 1996;**17**:1–12. doi:10.1016/0197-2456(95)00134-4

19 Sterne JAC, Savović J, Page MJ, *et al.* RoB 2: a revised tool for assessing risk of bias in randomised trials. *BMJ* 2019;**366**:l4898. doi:10.1136/bmj.l4898

For peer review only - http://bmjopen.bmj.com/site/about/guidelines.xhtml

20 Wells G, Shea B, O'Connell D, *et al.* The Newcastle-Ottawa Scale (NOS) for assessing the quality of nonrandomised studies in meta-analyses. Ottawa Hosp. Res. Inst.

21 Hong QN, Pluye P, Fàbregues S, *et al.* Mixed Methods Appraisal Tool (MMAT) Version 2018. McGIll Dep. Fam. Med. 2018.http://mixedmethodsappraisaltoolpublic.pbworks.com/w/file/fetch/127425851/MMAT_2018_criteria-manual_2018-04-04.pdf (accessed 15 Jul 2020).

22 Balshem H, Helfand M, Schünemann HJ, *et al.* GRADE guidelines: 3. Rating the quality of evidence. *J Clin Epidemiol* 2011;**64**:401–6. doi:10.1016/j.jclinepi.2010.07.015

23 PT Higgins J, Li T, K Deeks J. Chapter 6: Choosing effect measures and computing estimates of effect. In: *Cochrane Handbook for Systematic Reviews of Interventions version 6.0*. 2019. www.training.cochrane.org/handbook

For peer review only

For peer review only - http://bmjopen.bmj.com/site/about/guidelines.xhtml

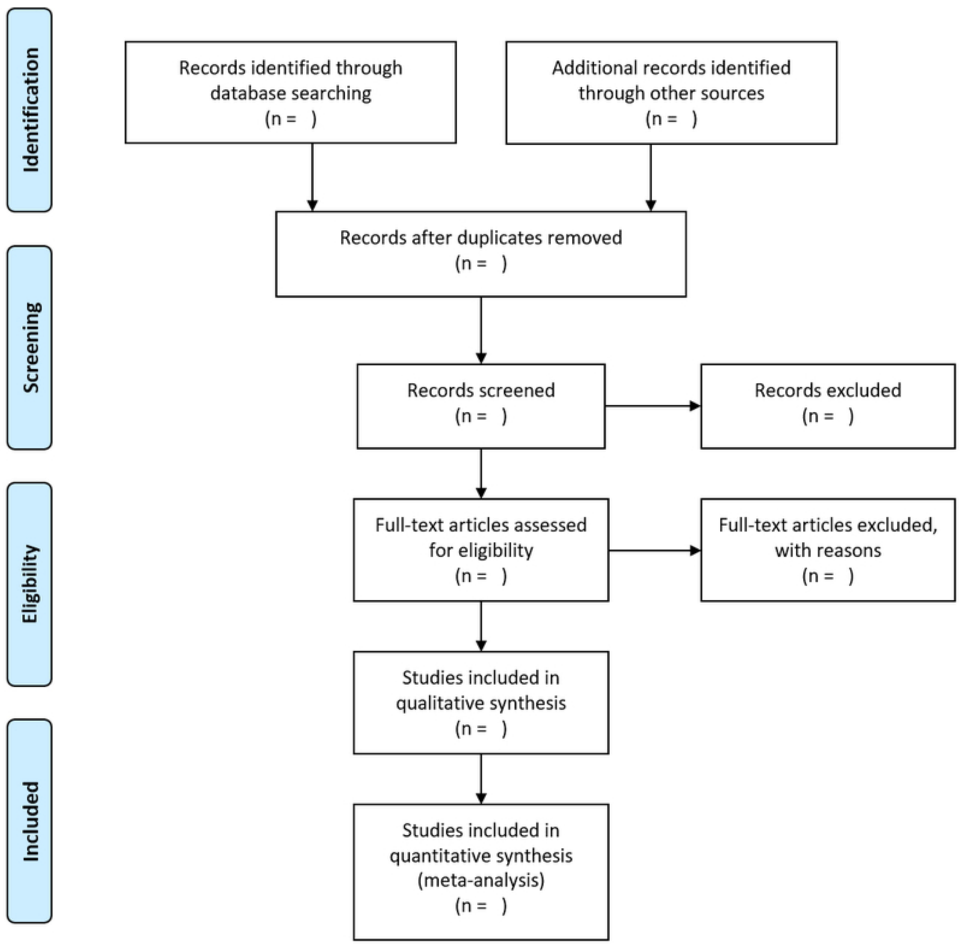

PRISMA Flowchart

70x66mm (300 x 300 DPI)

Appendix 1 – Example Medline search terms

Database: Medline (Ovid MEDLINE® Epub Ahead of Print, In-Process & Other Non-Indexed Citations, Ovid MEDLINE® Daily and Ovid MEDLINE®) 1946 to present

Search Strategy: (27/08/20)

----

1 exp Monitoring, Ambulatory/ (28543)

2 ambulatory.tw. (78329)

3 exp Wearable Electronic Devices/ (11822)

4 wearable.tw. (12014)

5 (body adj3 borne).tw. (72)

6 (head mount* adj3 (display* or device* or system*)).tw. (1101)

7 (head-up adj3 (display* or device* or system*)).tw. (153)

8 (head-worn adj3 (display* or device* or system*)).tw. (31)

9 (patch or patches).tw. (106471)

10 Adhesives/ (6682)

11 adhesive*.tw. (57587)

12 (sticker* or stick or sticks).tw. (12250)

13 (watch or watches).tw. (9028)

14 smartwatch*.tw. (309)

15 telemonitor*.tw. (1516)

16 tele monitor*.tw. (146)

17 exp Telemetry/ (13327)

18 telemet*.tw. (9378)

19 clothing/ or shoes/ (15121)

20 cloth*.tw. (16130)

For peer review only - http://bmjopen.bmj.com/site/about/guidelines.xhtml

21 exp Textiles/ (6392)

22 textile*.tw. (10792)

23 exp Telemedicine/ (29383)

24 telemedicine*.tw. (10894)

25 exp Biosensing Techniques/ (54829)

26 biosens*.tw. (37047)

27 glove*.tw. (10632)

28 shoe*.tw. (9295)

29 accessor*.tw. (44268)

30 Wrist/ (9011)

31 wrist*.tw. (38559)

32 necklace*.tw. (904)

33 (belt or belts).tw. (12916)

34 armband*.tw. (590)

35 (shirt or shirts).tw. (1067)

36 contactless.tw. (1731)

37 cableless.tw. (8)

38 contact sensor*.tw. (184)

39 unobtrusive.tw. (1596)

40 remote.tw. (69973)

41 Wireless Technology/ (3587)

42 wireless*.tw. (14654)

43 non contact.tw. (5281)

44 noncontact.tw. (4723)

45 without wires.tw. (21)

46 non-restrict*.tw. (555)

For peer review only

47 unrestrict*.tw. (8566)

48 Automation/ (18179)

49 automat*.tw. (229850)

50 webcam*.tw. (397)

51 web-cam*.tw. (175)

52 video*.tw. (126640)

53 2 or 3 or 4 or 5 or 6 or 7 or 8 or 9 or 10 or 11 or 12 or 13 or 14 or 15 or 16 or 17 or 18 or 19 or 20 or 21 or 22 or 23 or 24 or 25 or 26 or 27 or 28 or 29 or 30 or 31 or 32 or 33 or 34 or 35 or 36 or 37 or 38 or 39 or 40 or 41 or 42 or 43 or 44 or 45 or 46 or 47 or 48 or 49 or 50 or 51 or 52 (979503)

54 Monitoring, Physiologic/mt [Methods] (18136)

55 monitor*.tw. (804541)

56 Oximetry/ (13024)

57 oximet*.tw. (13397)

58 tracker*.tw. (4568)

59 Radar/ (1203)

60 radar*.tw. (5333)

61 Ballistocardiography/ (2049)

62 ballisto*.tw. (1831)

63 exp Accelerometry/ (8929)

64 accelerom*.tw. (16743)

65 gyro*.tw. (4773)

66 Photoplethysmography/ (1987)

67 ppg.tw. (3458)

68 photople*.tw. (3299)

69 videopleth*.tw. (4)

70 video pleth*.tw. (6)

For peer review only

71 (photo* adj3 pleth*).tw. (339)

72 Thermography/ (7571)

73 thermograph*.tw. (6133)

74 Infrared Rays/ (13281)

75 infrared*.tw. (137632)

76 thermal.tw. (200146)

77 thermistor*.tw. (1748)

78 thermoresister*.tw. (0)

79 camera*.tw. (45491)

80 wavelet analysis/ (2189)

81 wavelet*.tw. (11286)

82 waveform*.tw. (28510)

83 ambient light*.tw. (1886)

84 piezoelectric*.tw. (10492)

85 piezo electric*.tw. (342)

86 Electric Impedance/ (17145)

87 impedan*.tw. (45070)

88 exp Electrocardiography/ (204339)

89 electrocar*.tw. (88334)

90 (ecg or ecgs).tw. (65572)

91 (ekg or ekgs).tw. (3081)

92 electrom*.tw. (97510)

93 electrog*.tw. (20290)

94 exp Electrodes/ (130099)

95 electrode*.tw. (161247)

96 SensiumVitals.tw. (8)

For peer review only

For peer review only - http://bmjopen.bmj.com/site/about/guidelines.xhtml

97 IntelliVue Guardian.tw. (0)

98 VitalPatch.tw. (2)

99 Vitalsolutions.tw. (0)

100 Guardian Angel.tw. (74)

101 Visi Mobile.tw. (6)

102 Current health.tw. (4290)

103 Snap40.tw. (1)

104 Multi-vital ECG patch.tw. (0)

105 Caretaker.tw. (1456)

106 Life scope G3.tw. (0)

107 Biostamp.tw. (4)

108 TAGECG.tw. (0)

109 ZioXT patch.tw. (0)

110 ZioAT patch.tw. (0)

111 Cardea solo.tw. (0)

112 Peerbridge Cor.tw. (0)

113 Radius-7.tw. (24)

114 Radius-PPG.tw. (0)

115 WristOx2.tw. (4)

116 Lifesync.tw. (1)

117 Ariatele.tw. (0)

118 54 or 55 or 56 or 57 or 58 or 59 or 60 or 61 or 62 or 63 or 64 or 65 or 66 or 67 or 68 or 69 or 70 or 71 or 72 or 73 or 74 or 75 or 76 or 77 or 78 or 79 or 80 or 81 or 82 or 83 or 84 or 85 or 86 or 87 or 88 or 89 or 90 or 91 or 92 or 93 or 94 or 95 or 96 or 97 or 98 or 99 or 100 or 101 or 102 or 103 or 104 or 105 or 106 or 107 or 108 or 109 or 110 or 111 or 112 or 113 or 114 or 115 or 116 or 117 (1802638)

119 53 and 118 (177463)

120 1 or 119 (194037)

For peer review only - http://bmjopen.bmj.com/site/about/guidelines.xhtml

121 vital signs/ or blood pressure/ or body temperature/ or heart rate/ or respiratory rate/ (410477)

122 heart rate*.tw. (159199)

123 pulse rate*.tw. (7614)

124 pulse*.tw. (256042)

125 respiratory rate*.tw. (14992)

126 breathing rate*.tw. (1234)

127 respiration.tw. (67094)

128 breathing.tw. (71424)

129 blood pressure.tw. (294342)

130 systolic.tw. (168584)

131 diastolic.tw. (124777)

132 perfusion.tw. (159943)

133 spo2.tw. (5098)

134 oxygenation.tw. (52011)

135 oxygen saturation.tw. (26170)

136 temperature*.tw. (651316)

137 exp Fever/ (43448)

138 fever*.tw. (171789)

139 hypertherm*.tw. (35327)

140 pyrex*.tw. (5346)

141 Hypothermia/ (13917)

142 hypotherm*.tw. (41950)

143 apyrex*.tw. (210)

144 a-pyrex*.tw. (173)

145 vital sign*.tw. (14891)

146 vitals.tw. (500)

For peer review only

For peer review only - http://bmjopen.bmj.com/site/about/guidelines.xhtml

147 vital param*.tw. (1004)

148 vital function*.tw. (2790)

149 early warning score/ (91)

150 (early warning adj3 scor*).tw. (1049)

151 (early warning adj3 system*).tw. (2134)

152 mews.tw. (261)

153 ews.tw. (1986)

154 ewss.tw. (73)

155 (track and trigger).tw. (331)

156 tts.tw. (2485)

157 risk assessment tool*.tw. (3049)

158 febrile.tw. (35432)

159 afebrile.tw. (2897)

160 a-febrile.tw. (2540)

161 121 or 122 or 123 or 124 or 125 or 126 or 127 or 128 or 129 or 130 or 131 or 132 or 133 or 134 or 135 or 136 or 137 or 138 or 139 or 140 or 141 or 142 or 143 or 144 or 145 or 146 or 147 or 148 or 149 or 150 or 151 or 152 or 153 or 154 or 155 or 156 or 157 or 158 or 159 or 160 (2068958)

162 Hospitalization/ (108262)

163 hospital*.tw. (1288499)

164 inhospital.tw. (1750)

165 in-hospital.tw. (89036)

166 admitted.tw. (206110)

167 inpatient*.tw. (108524)

168 surgical.tw. (968814)

169 pacu.tw. (2502)

170 post anaesthesia care unit*.tw. (328)

For peer review only - http://bmjopen.bmj.com/site/about/guidelines.xhtml

171 post anesthesia care unit*.tw. (740)

172 postanaesthesia care unit*.tw. (192)

173 postanesthesia care unit*.tw. (1900)

174 icu.tw. (56820)

175 exp Critical Care/ (58129)

176 exp Intensive Care Units/ (85074)

177 intensive care.tw. (144066)

178 itu.tw. (869)

179 aicu.tw. (45)

180 icus.tw. (10393)

181 itus.tw. (43)

182 aicus.tw. (7)

183 pacus.tw. (82)

184 critical care.tw. (27729)

185 (ward or wards).tw. (59542)

186 Triage/ (11887)

187 triage*.tw. (18767)

188 Emergencies/ (40466)

189 (emergency or emergencies).tw. (270872)

190 Recovery Room/ (1297)

191 recovery room*.tw. (3323)

192 adolescent, hospitalized/ or child, hospitalized/ or inpatients/ (29074)

193 162 or 163 or 164 or 165 or 166 or 167 or 168 or 169 or 170 or 171 or 172 or 173 or 174 or 175 or 176 or 177 or 178 or 179 or 180 or 181 or 182 or 183 or 184 or 185 or 186 or 187 or 188 or 189 or 190 or 191 or 192 (2563179)

194 120 and 161 and 193 (5355)

For peer review only

For peer review only - http://bmjopen.bmj.com/site/about/guidelines.xhtml

For peer review only

For peer review only - http://bmjopen.bmj.com/site/about/guidelines.xhtml

PRISMA-P (Preferred Reporting Items for Systematic review and Meta-Analysis Protocols) 2015 checklist: recommended items to address in a systematic review protocol*

Section and topic	Item No	Checklist item	Page
**ADMINISTRATIVE INFORMATION**			
Title:			
Identification	1a	Identify the report as a protocol of a systematic review	1
Update	1b	If the protocol is for an update of a previous systematic review, identify as such	N/A
Registration	2	If registered, provide the name of the registry (such as PROSPERO) and registration number	2
Authors:			
Contact	3a	Provide name, institutional affiliation, e-mail address of all protocol authors; provide physical mailing address of corresponding author	1
Contributions	3b	Describe contributions of protocol authors and identify the guarantor of the review	11
Amendments	4	If the protocol represents an amendment of a previously completed or published protocol, identify as such and list changes; otherwise, state plan for documenting important protocol amendments	11
Support:			
Sources	5a	Indicate sources of financial or other support for the review	11
Sponsor	5b	Provide name for the review funder and/or sponsor	11
Role of sponsor or funder	5c	Describe roles of funder(s), sponsor(s), and/or institution(s), if any, in developing the protocol	11
**INTRODUCTION**			
Rationale	6	Describe the rationale for the review in the context of what is already known	3
Objectives	7	Provide an explicit statement of the question(s) the review will address with reference to participants, interventions, comparators, and outcomes (PICO)	4
**METHODS**			
Eligibility criteria	8	Specify the study characteristics (such as PICO, study design, setting, time frame) and report characteristics (such as years considered, language, publication status) to be used as criteria for eligibility for the review	4-6
Information sources	9	Describe all intended information sources (such as electronic databases, contact with study authors, trial registers or other grey literature sources) with planned dates of coverage	6
Search strategy	10	Present draft of search strategy to be used for at least one electronic database, including planned limits, such that it could be repeated	Appendix 1

For peer review only

Study records:			
Data management	11a	Describe the mechanism(s) that will be used to manage records and data throughout the review	7
Selection process	11b	State the process that will be used for selecting studies (such as two independent reviewers) through each phase of the review (that is, screening, eligibility and inclusion in meta-analysis)	7
Data collection process	11c	Describe planned method of extracting data from reports (such as piloting forms, done independently, in duplicate), any processes for obtaining and confirming data from investigators	7
Data items	12	List and define all variables for which data will be sought (such as PICO items, funding sources), any pre-planned data assumptions and simplifications	7
Outcomes and prioritization	13	List and define all outcomes for which data will be sought, including prioritization of main and additional outcomes, with rationale	5-6
Risk of bias in individual studies	14	Describe anticipated methods for assessing risk of bias of individual studies, including whether this will be done at the outcome or study level, or both; state how this information will be used in data synthesis	8-9
Data synthesis	15a	Describe criteria under which study data will be quantitatively synthesised	9
	15b	If data are appropriate for quantitative synthesis, describe planned summary measures, methods of handling data and methods of combining data from studies, including any planned exploration of consistency (such as I^2, Kendall's τ)	9
	15c	Describe any proposed additional analyses (such as sensitivity or subgroup analyses, meta-regression)	9
	15d	If quantitative synthesis is not appropriate, describe the type of summary planned	9
Meta-bias(es)	16	Specify any planned assessment of meta-bias(es) (such as publication bias across studies, selective reporting within studies)	9
Confidence in cumulative evidence	17	Describe how the strength of the body of evidence will be assessed (such as GRADE)	9

*** It is strongly recommended that this checklist be read in conjunction with the PRISMA-P Explanation and Elaboration (cite when available) for important clarification on the items. Amendments to a review protocol should be tracked and dated. The copyright for PRISMA-P (including checklist) is held by the PRISMA-P Group and is distributed under a Creative Commons Attribution Licence 4.0.**

From: Shamseer L, Moher D, Clarke M, Ghersi D, Liberati A, Petticrew M, Shekelle P, Stewart L, PRISMA-P Group. Preferred reporting items for systematic review and meta-analysis protocols (PRISMA-P) 2015: elaboration and explanation. BMJ. 2015 Jan 2;349(jan02 1):g7647.

For peer review only
